# The Diagnostic Challenge of Rapunzel Syndrome: Multimodal Imaging Findings in a Child

**DOI:** 10.3390/diagnostics16060940

**Published:** 2026-03-22

**Authors:** Chih-Hao Wang, Shu-Chao Weng

**Affiliations:** 1 Department of Pediatrics, MacKay Children’s Hospital, Taipei 104217, Taiwan; mmh4972@gmail.com; 2 Department of Pediatric Gastroenterology, Hepatology and Nutrition, MacKay Children’s Hospital, Taipei 104217, Taiwan; 3 Department of Medicine, Mackay Medical University, New Taipei City 252005, Taiwan

**Keywords:** abdominal mass, trichobezoar, ultrasonography, computed tomography, endoscopy, Rapunzel syndrome

## Abstract

We report the case of a school-aged patient with attention-deficit/hyperactivity disorder who presented with a palpable epigastric mass. The initial abdominal ultrasonography indicated the presence of a heterogeneous space-occupying lesion in the upper abdomen. Subsequent computed tomography revealed a large intragastric mass with a mottled air-containing density, an imaging feature characteristic of a bezoar. Esophagogastroduodenoscopy confirmed a massive trichobezoar extending beyond the pylorus into the duodenum, consistent with Rapunzel syndrome. Although the endoscopic removal proved unsuccessful, a subsequent surgical extraction via laparotomy yielded a 22 cm trichobezoar. Further history revealed prior hair-picking behavior approximately one year earlier, with a localized bald patch noted by her parents. This case highlights the characteristic multimodal imaging findings of trichobezoars and serves to emphasize the diagnostic challenge posed by a limited clinical history in children with psychiatric comorbidities presenting with abdominal masses.

**Figure 1:**
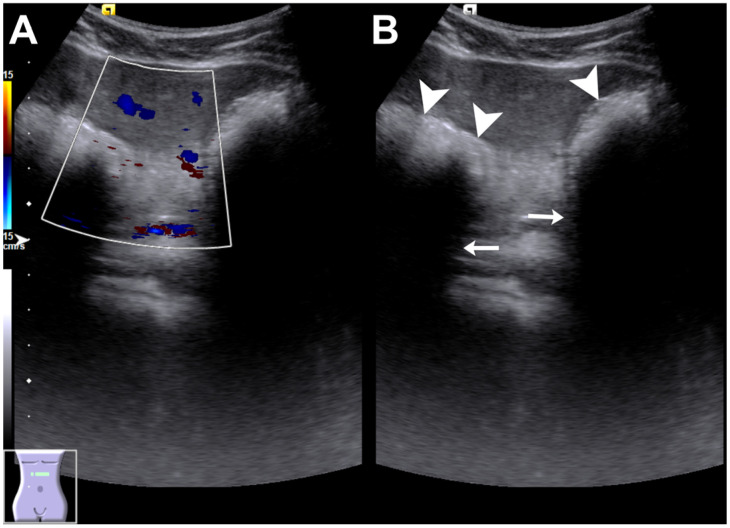
Abdominal ultrasonography of a gastric trichobezoar. A school-aged patient with attention-deficit/hyperactivity disorder was found to have a firm upper abdominal mass during a routine health examination. She reported early satiety but denied abdominal pain, vomiting, or weight loss. An abdominal examination revealed a well-defined, firm, non-tender mass measuring approximately 10 × 5 cm across the epigastrium, with a smooth surface and slight mobility on respiration. No hepatosplenomegaly or tenderness was noted, and the laboratory findings were unremarkable. Abdominal ultrasonography revealed a heterogeneous hyperechoic intragastric mass without internal vascularity on color Doppler imaging (**A**) and a markedly hyperechoic intraluminal interface (arrowheads) with prominent posterior acoustic shadowing (arrows) (**B**), which are considered sonographic features characteristic of a gastric bezoar. Upon further inquiry, her parents reported that she had exhibited hair-picking behavior approximately one year earlier, with a localized bald area noted at that time. In clinical practice, abdominal ultrasonography serves as an initial screening and clue-providing tool. Previous reports describe gastric trichobezoars on ultrasonography as well-defined intragastric hyperechoic masses with marked posterior acoustic attenuation or shadowing, sometimes initially mistaken for calcified lesions. In some cases, a characteristic a “hyperechoic ecoattenuating mass bordered by a trilinear wall” configuration has been noted. These lesions are most commonly located in the epigastric region corresponding to the stomach, although small bowel bezoars have also been reported [1,2,3]. The recognition of these characteristic patterns could prompt diagnostic confirmation via endoscopy and an assessment of the disease extent or complications based on computed tomography (CT), particularly in pediatric and young female patients with a palpable epigastric mass [4].

**Figure 2:**
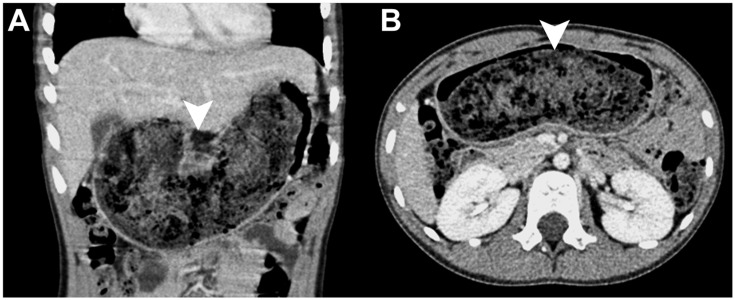
Contrast-enhanced computed tomography of the gastric trichobezoar. The contrast-enhanced abdominal CT demonstrates a large intragastric lesion with a heterogeneous, air-mixed density (arrowheads), consistent with bezoar formation, shown on coronal (**A**) and axial (**B**) images. Previous reports describe gastric trichobezoars on CT as well-defined intraluminal masses with a characteristic mottled appearance caused by numerous entrapped air bubbles within the tightly woven hairs. The lesion typically fills the gastric body and fundus and may extend beyond the pylorus into the duodenum or small bowel in Rapunzel syndrome or appear as separate small bowel masses [2,3,5,6]. Accordingly, abdominal CT is considered the single most effective modality for staging trichobezoars, enabling the assessment of the lesion extent, small bowel involvement, subsidiary bezoars, and obstruction or ischemic risk; evaluations of all are essential for surgical planning [3,7].

**Figure 3:**
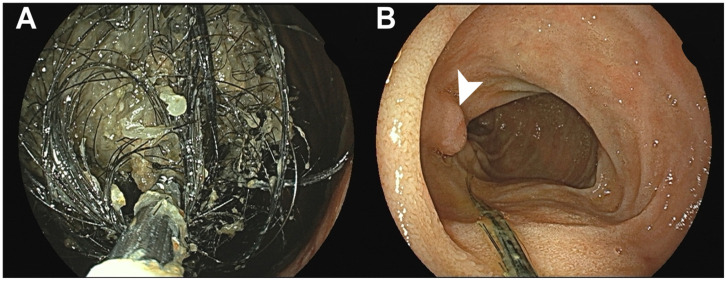
The endoscopic appearance of the gastric trichobezoar. The patient was instructed to consume Coca-Cola^®^ (The Coca-Cola Company, Atlanta, GA, USA) for several days in an attempt to facilitate bezoar dissolution. Esophagogastroduodenoscopy (EGD) revealed (**A**) a massive hair-containing bezoar occupying the gastric lumen and (**B**) the extension of the bezoar into the second portion of the duodenum, consistent with Rapunzel syndrome. The arrowhead indicates the ampulla of Vater. Additional Coca-Cola^®^ was injected endoscopically, and partial fragmentation was attempted using forceps; however, complete removal was unsuccessful. EGD is considered the gold standard for diagnosing gastric trichobezoars because it allows for the direct visualization of the mass and its composition [5]. Previous reports have described a large intragastric mass composed of compacted hair occupying the gastric body and fundus, sometimes extending into the duodenal bulb or beyond in cases of Rapunzel syndrome [6,8,9]. In cases of mixed trichophytobezoars, EGD may reveal a densely packed hair ball with a firm core containing vegetable fibers [10]. The surrounding gastric mucosa may demonstrate erosions, non-bleeding ulcers, or polypoid changes attributed to chronic mechanical pressure and friction from the bezoar [4,11,12]. Furthermore, Coca-Cola^®^ dissolution therapy has been widely reported as an effective and minimally invasive treatment for phytobezoars. Its proposed mechanism involves acidity (pH ~2.6), which mimics gastric acid and helps break down plant fibers, as well as the mucolytic effect of sodium bicarbonate and the mechanical action of carbon dioxide bubbles that loosen and fragment the fiber–mucus matrix. Reported overall success rates exceed 90% when combined with endoscopic fragmentation; however, these data pertain primarily to gastric phytobezoars and do not include trichobezoars [13]. Trichobezoars are composed of tightly packed hair made of keratin and lack the cellulose and tannin matrix present in phytobezoars [10,13,14]. Keratin is highly resistant to chemical dissolution, and available evidence from case reports and reviews indicates that Coca-Cola^®^ does not reliably dissolve gastric trichobezoars and is at best a minor adjunct before eventual endoscopic or surgical removal [5,9,14]. Therefore, Coca-Cola^®^ therapy is not considered a standard treatment for trichobezoars and has been reported as a successful intervention in only rare cases [9]. In the present case, Coca-Cola^®^ was administered as an initial, minimally invasive attempt prior to a more definitive intervention, acknowledging its limited expected efficacy in hair-based bezoars.

**Figure 4:**
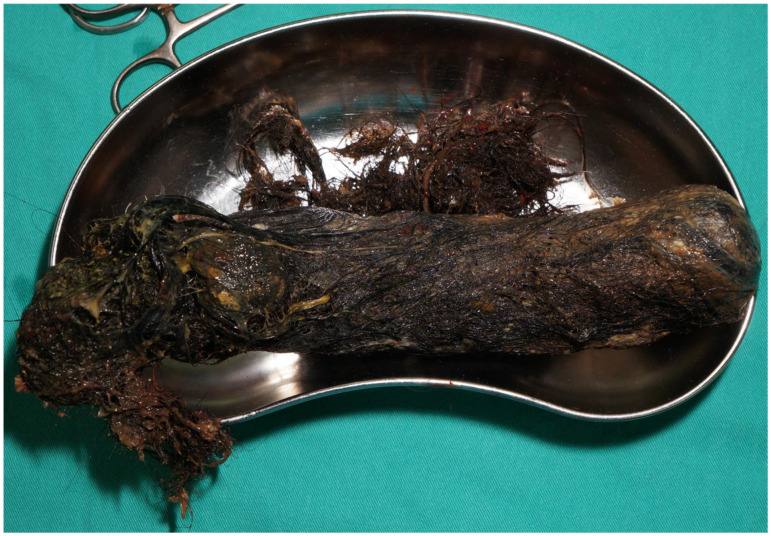
The gross specimen following surgical removal. A subsequent laparotomy with gastrostomy was performed to remove a 22 cm trichobezoar. The postoperative course was uneventful, and the patient was discharged with a planned psychiatric follow-up. Trichobezoars are rare gastric foreign bodies that typically affect young females with psychiatric comorbidities. They are strongly associated with trichotillomania and trichophagia, often occurring in the context of emotional distress or other psychological disorders. In pediatric patients, these behaviors may be underrecognized or underreported, delaying diagnosis and allowing progressive hair accumulation [4,5]. Common presentations include early satiety, abdominal pain, or a palpable mass [15]. Abdominal masses in children may originate from multiple structures, including the gastrointestinal tract, liver, pancreas, kidneys, adrenal glands, and mesentery, and encompass a broad range of congenital, inflammatory, and neoplastic entities [16]. Ultrasonography is typically the first-line imaging modality to confirm and characterize a suspected mass, with CT or magnetic resonance imaging often used for the further delineation of its origin and internal architecture [17]. In the present case, the intraluminal location and characteristic imaging findings, including marked posterior acoustic shadowing on ultrasonography and a mottled air-containing mass on CT, strongly favored the diagnosis of a bezoar rather than cystic or solid intra-abdominal masses. In addition, the management of trichobezoars is primarily determined by the size, extent, and the presence of complications [4,5,8,15]. As discussed above, chemical dissolution plays only a limited adjunctive role in trichobezoars due to their keratin composition [5,9,14]. Endoscopic removal may be feasible in small, localized gastric trichobezoars, particularly when confined to the stomach and without evidence of obstruction or perforation [12]. Surgical removal remains the standard of care for large, firm, or Rapunzel-type trichobezoars, especially when extension beyond the pylorus is present [8]. Laparoscopic or mini-laparotomy approaches have been successfully utilized as minimally invasive alternatives that offer advantages such as better cosmetic results and shorter hospital stays, while an open laparotomy is generally preferred for giant/large bezoars, duodenal or jejunal extension, or suspected complications [5,8,15]. Regardless of the removal method, psychiatric evaluation and structured follow-up are essential to address underlying trichotillomania or trichophagia and to prevent recurrence [4,6,8]. In this minimally symptomatic child, careful physical examination, supported by characteristic ultrasonographic and computed tomography findings, enabled the timely endoscopic confirmation of a trichobezoar, despite a limited and often undisclosed patient history [18].

## Data Availability

No new data were created or analyzed in this study. Data sharing is not applicable to this article.

## Abbreviations

The following abbreviations are used in this manuscript:

CT	Computed tomography
EGD	Esophagogastroduodenoscopy
